# MicroRNAs in Cancer Immunology: Master Regulators of the Tumor Microenvironment and Immune Evasion, with Therapeutic Potential

**DOI:** 10.3390/cancers17132172

**Published:** 2025-06-27

**Authors:** Erfan Zare, Seyyed Mohammad Yaghoubi, Maedeh Khoshnazar, Sina Jafari Dargahlou, Janvhi Suresh Machhar, Zihan Zheng, Pascal H. G. Duijf, Behzad Mansoori

**Affiliations:** 1 Cancer Immunology and Immunotherapy Research Center, Ardabil University of Medical Sciences, Ardabil 5371713111, Iran; er.zare79@gmail.com; 2 Students Research Committee, School of Medicine, Ardabil University of Medical Sciences, Ardabil 5618985991, Iran; 3 Department of Plant, Cell and Molecular Biology, Faculty of Natural Sciences, University of Tabriz, Tabriz 5166616471, Iran; myardir@gmail.com; 4 Department of Developmental Biology, TeMS.C. Islamic Azad University, Tehran 18116-94784, Iran; maedehkhoshnazar2005@gmail.com; 5 Department of Biophysics, Faculty of Advanced Technologies, University of Mohaghegh Ardabili, Namin 5619911367, Iran; sina.jafari.428@gmail.com; 6 The Wistar Institute, Molecular & Cellular Oncogenesis Program, Philadelphia, PA 19036, USA; jmachhar@wistar.org (J.S.M.); zzheng@wistar.org (Z.Z.); 7 Centre for Cancer Biology, Clinical and Health Sciences, University of South Australia & SA Pathology, Adelaide, SA 5001, Australia; pascal.duijf@unisa.edu.au; 8 School of Biomedical Sciences, Faculty of Health, Queensland University of Technology, Brisbane, QLD 4102, Australia

**Keywords:** miRNA, cancer, immunology, tumor microenvironment, evasion, surveillance, equilibrium, immunometabolism

## Abstract

MicroRNAs (miRNAs) are pivotal modulators of tumor progression and immune function. Given the central role of the immune system in recognizing and eliminating malignant cells, understanding how miRNAs influence immune responses has become essential for advancing cancer therapy. This review explores the emerging roles of miRNAs in orchestrating cancer immunology, emphasizing their regulation of tumor immune surveillance, immune equilibrium, immune evasion, and immunometabolism. We further illustrate how specific miRNAs modulate the tumor microenvironment by shaping immune cell phenotypes, cytokine networks, and antigen presentation. Some miRNAs enhance cytotoxic T lymphocyte activity, while others promote immune escape by expanding regulatory T cells and myeloid-derived suppressor cells. miRNAs also regulate immune checkpoints (e.g., PD-L1 and CTLA-4), metabolic reprogramming, and stress responses that collectively influence tumor immunogenicity. Additionally, miRNAs are gaining traction as biomarkers for immune activity and predictors of immunotherapy response. Therapeutically, miRNA mimics and inhibitors can enhance anti-tumor immunity, particularly when combined with advanced delivery platforms or immune checkpoint inhibitors. However, challenges such as delivery specificity, off-target effects, and the context-dependent nature of miRNA activity remain significant barriers to clinical translation. Despite shortcomings, miRNAs represent a class of immune regulators with substantial therapeutic potential. Accelerated progress in miRNA-guided therapies is anticipated through deepening insights into miRNA regulatory networks, coupled with integrative multi-omics and AI-driven analytical frameworks. Altogether, miRNAs are a promising frontier in next-generation cancer immunotherapy and precision oncology.

## Simple Summary

MicroRNAs are small molecules that help control how genes are turned on or off. In cancer, they play a key role not only in how tumors grow but also in how the immune system responds to cancer. Some microRNAs help the immune system attack tumors, while others help cancer cells hide from immune defenses. This review looks at how microRNAs affect different parts of the immune response to cancer, including immune cell behavior, inflammation, and how the body recognizes cancer cells. We also explore how microRNAs might be used to predict which patients will respond to immunotherapy and how they could become part of new treatments. Although challenges remain, such as making sure microRNA-based drugs reach the right cells, this area of research has great promise. Understanding these small molecules could lead to more effective and personalized cancer therapies in the future.

## 1. Introduction

MicroRNAs (miRNAs) are a class of small, non-coding RNA molecules, approximately 19–25 nucleotides in length, that mediate post-transcriptional regulation of gene expression. First identified nearly two decades ago, miRNAs exert their effects primarily through base-pairing with complementary sequences in the 3′ untranslated regions (3′-UTRs) of target messenger RNAs (mRNAs), resulting in translational repression or mRNA degradation [1,2]. The biogenesis of miRNAs is a highly orchestrated, multistep process. It begins with transcription, followed by nuclear processing via the Drosha-DGCR8 microprocessor complex, cytoplasmic cleavage by Dicer, and subsequent incorporation of mature miRNA duplexes into RNA-induced silencing complexes (RISCs), which mediate gene silencing through sequence-specific interactions with target mRNAs [3]. Functionally, miRNAs play a fundamental role in regulating a broad spectrum of cellular and physiological processes, including cell fate determination, proliferation, apoptosis, metabolic homeostasis, and intercellular signaling. Through modulation of gene regulatory networks, microRNAs control the expression of hundreds of protein-coding genes involved in critical cellular functions, including cell cycle progression, apoptosis, immune responses, metabolic pathways, etc. Estimates suggest that over 60% of human protein-coding genes are under direct or indirect regulation by microRNAs, highlighting their widespread influence on gene expression and their roles in both physiological processes and disease development. The immune system is integral to maintaining organismal integrity by identifying and eliminating aberrant or transformed cells, including malignant cells. Under physiological conditions, immune surveillance enables the detection and eradication of emerging neoplastic cells, thereby preventing tumor initiation and progression [4,5]. Nevertheless, tumor cells can evolve diverse mechanisms to escape immune recognition and destruction, contributing to immune evasion and cancer development. Elucidating the molecular pathways underlying these immune escape mechanisms is critical for the advancement of effective immunotherapeutic strategies. Accumulating evidence indicates that miRNAs are key modulators of immune cell development, differentiation, and function within the tumor microenvironment (TME). Aberrant expression of miRNAs in both tumor cells and infiltrating immune cells can significantly influence the immune landscape, altering the efficacy of anti-tumor responses and facilitating tumor immune escape [6,7,8].

This review aims to provide a comprehensive overview of the role of miRNAs in various aspects of cancer immunology, including tumor immune surveillance, immune equilibrium, immune evasion, and immunometabolism. We also discuss how a mechanistic understanding of these regulatory networks informs the development of miRNA-based therapies to enhance anti-cancer immunity.

## 2. miRNAs and Immune Surveillance

**Figure 1:**
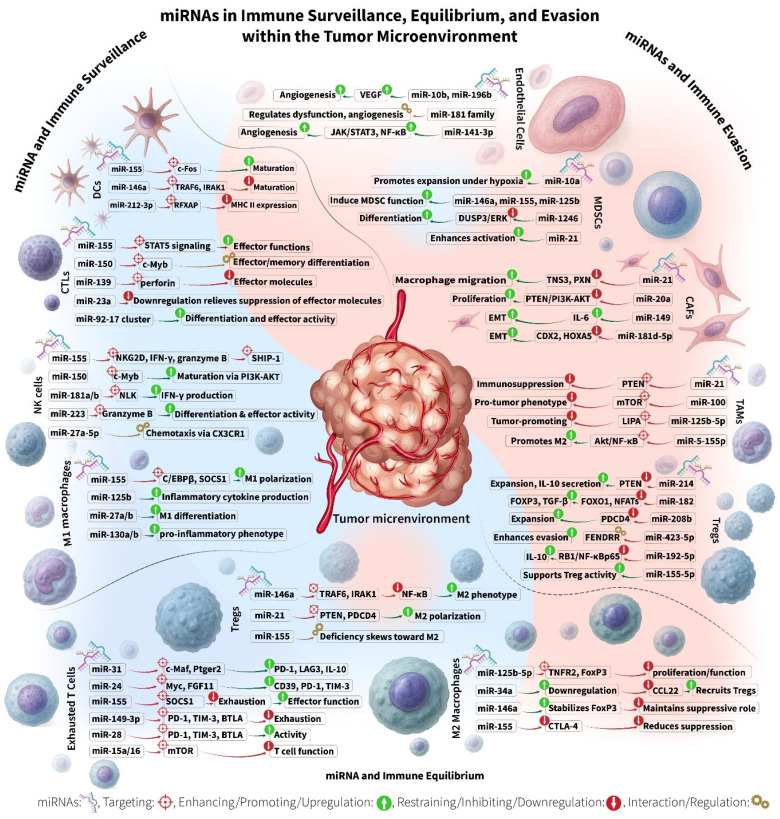
The roles of miRNAs in immune surveillance, equilibrium, and evasion within the TME. miRNAs can regulate functions of immune cell populations, including CTLs, NK, DCs, TAMs, MDSCs, and Tregs in TME through different pathways.

In the TME, miRNAs have emerged as key regulators of immune surveillance by modulating processes such as antigen presentation, costimulatory signaling, and T cell priming [9]. These small non-coding RNAs dynamically regulate the expression of genes involved in the activation and function of multiple immune cell populations. For instance, miRNA-mediated downregulation of antigen-processing machinery or MHC class I molecules in tumor cells and dendritic cells (DCs) can impair antigen presentation, limiting recognition of tumor antigens by cytotoxic T lymphocytes (CD8^+^ T cells) (Table 1 and Figure 1) [10].

Similarly, miRNAs can target costimulatory ligands or cytokines necessary for full T cell activation, thus altering the priming of helper T cells (CD4^+^ T cells) and subsequent adaptive responses [136]. By shaping these key signals, miRNAs indirectly dictate the efficacy of cytotoxic CD8^+^ T cell responses, CD4^+^ helper T cell polarization, and natural killer (NK) cell-mediated cytotoxicity [137]. Furthermore, miRNAs influence the behavior of innate immune cells in the TME. For example, they can polarize tumor-associated macrophages (TAM) toward immunosuppressive or immunostimulatory phenotypes [138,139]. They also modulate tumor-associated neutrophils (TAN), impacting these cells’ ability to either dampen or promote anti-tumor immunity [140]. Understanding these multifaceted roles of miRNAs in orchestrating immune surveillance highlights their importance in tipping the balance between tumor elimination and immune evasion. This underscores their potential as targets for cancer immunotherapy. In the following sections, we will review the roles of miRNAs in regulating immune surveillance within the TME by focusing on their impact on individual immune cell populations, including cytotoxic T lymphocytes, NK cells, DCs, TAMs, and TAN.

### 2.1. T Lymphocytes

T lymphocytes are central to cancer immune surveillance, acting as key effectors of the adaptive immune system that can recognize and eliminate malignant cells. Several T cell subsets contribute to this process, each with distinct roles [141].

### 2.2. CD8^+^ Cytotoxic T Lymphocytes (CTLs)

miRNAs are critical regulators of CTL differentiation, activation, cytotoxicity, and exhaustion in the context of cancer (Table 1 and Figure 1). For instance, miR-23a suppresses CTL effector functions; it is downregulated during DC priming, thereby enhancing cytotoxicity. Overexpression of miR-23a impairs effector molecule expression, whereas its inhibition mitigates TGF-β-induced immunosuppression and restores granzyme B levels and thereby cytotoxic T cell function [11]. Similarly, miR-139 inhibits perforin expression, and its downregulation during inflammation enhances CTL cytotoxicity [15]. miR-150 modulates the differentiation of naïve CD8^+^ into effector and memory subsets via regulation of C-Myb [15,16]. miR-155 is essential for CTL responses in both cancer and viral infections, partly through its role in interferon signaling [19].

Other miRNAs, including miR-10a-5p and miR-135b, influence cytotoxic molecule expression. For example, miR-135b silencing reduces granzyme B and perforin 1 in anaplastic large cell lymphoma cells, implicating it in CTL-mediated cytotoxicity [30,31].

CTL differentiation and memory formation are also miRNA-dependent. Loss of Dicer, a key enzyme in miRNA processing, disrupts T cell development and impairs antigen-specific CTL responses [142]. The miR-17-92 cluster and miR-155 regulate CTL differentiation during antiviral and antitumor responses, while miR-150 influences the fate of central memory CD8^+^ [15].

miRNAs can also influence tumor–CTL interactions. For instance, Dicer deficiency in tumor cells leads to ICAM-1 upregulation, enhancing CTL–tumor cell conjugation and antigen-specific cytotoxicity. Conversely, EBV-encoded miRNAs inhibit recognition and killing of infected cells by downregulating HLA class I molecules [143].

Within the TME, miRNAs further modulate CTL function. TGF-β upregulates miR-23a in tumor-infiltrating lymphocytes (TILs), suppressing effector molecules and promoting immune evasion [11].

### 2.3. CD4^+^ Helper T Lymphocytes

miRNAs also regulate CD4^+^ T cell differentiation and function, shaping the polarization of Th1, Th2, Th17, and Treg (Regulatory T Cell) subsets (Table 1 and Figure 1). These changes in T helper cell responses can indirectly affect CTL responses and tumor immunity. For example, miR-155 promotes Th1 differentiation, while its absence shifts T cells toward a Th2 phenotype [20]. miR-326 facilitates Th17 development, and the miR-17-92 cluster supports T follicular helper (Tfh) cell function [32,33]. In contrast, miR-23 and miR-27 inhibit Treg/Th17 and Th1 differentiation, while miR-24 promotes these phenotypes, contributing to an immunosuppressive TME [144,145].

Several miRNAs also influence cytokine production, thereby influencing CD4^+^ T cell polarization. miR-107, for example, targets IL-23p19 in DC, potentially altering T cell polarization [37]. miR-135b enhances IL-17 production by repressing Th2 regulators STAT6 and GATA3; its inhibition reduces expression of IL-17A, IL-17F, IL-6, and IL-8 [30].

The balance between Th cell subsets is critical for anti-tumor immunity. A Th1-skewed response typically favors tumor control. Tumor-derived exosomes carrying miRNAs can modulate dendritic cell maturation and thereby influence CD4^+^ T cell differentiation. For example, exosomal miR-let-7i can suppress immune responses by altering cytokine expression in DC [38].

### 2.4. Regulatory T Cells (Treg)

Tregs suppress anti-tumor immunity and rely on miRNAs for their development, maintenance, and function (Table 1 and Figure 1). Dicer-dependent miRNA biogenesis is essential for Treg-mediated immune homeostasis; its loss results in lethal inflammation. For instance, miR-192-5p promotes Treg differentiation by regulating IL-10 [40].

In the TME, tumor-derived exosomes can induce neoplastic Tregs, supporting immune evasion, while Treg-derived miRNAs modulate dendritic cell activity, reinforcing immunosuppression. Treg recruitment and function are influenced by TGF-β-induced miRNA expression, further suppressing effector T cell activity and blunting anti-tumor immunity [146]. For instance, TGF-β can influence Treg recruitment by suppressing miR-34a, leading to increased CCL22 production [42]. Since TGF-β is active in the TME, miR-214 may also be upregulated, further contributing to Treg expansion. Tumor-derived miR-214 is transported to CD4^+^ T cells, where it promotes Treg expansion by downregulating expression of the PTEN tumor suppressor [41].

Taken together, miRNAs orchestrate the differentiation, function, and fate of T lymphocyte subsets central to cancer immune surveillance. They target key signaling pathways and effector molecules in both immune cells and the TME, thereby either potentiating or inhibiting anti-tumor immunity. Immunosuppressive miRNAs often facilitate tumor progression and resistance to immunotherapy by dampening CTL function and enhancing Treg activity. In contrast, miRNAs that support CTL cytotoxicity and Th1 responses may improve patient responses to immunotherapies, including checkpoint blockade. A deeper understanding of miRNA-mediated regulation in T cells offers promising avenues for designing targeted immunotherapeutic strategies aimed at enhancing anti-tumor immunity and improving clinical outcomes.

### 2.5. Natural Killer (NK) Cells

Natural killer (NK) cells serve as a critical first line of defense in cancer immune surveillance. As innate lymphocytes, they recognize and eliminate transformed cells by detecting downregulated MHC class I molecules or upregulated stress ligands on tumor cells. Upon activation, NK cells release cytotoxic granules containing perforin and granzymes, leading to target cell death [147]. They also secrete cytokines and chemokines that shape broader immune responses. NK cell activity is finely tuned by a balance of activating receptors, such as NKG2D, and inhibitory receptors that detect self-MHC molecules [147]. miRNAs are key regulators of NK cell development, maturation, cytotoxicity, and tumor recognition (Table 1 and Figure 1).

Several miRNAs modulate NK cell cytotoxicity by targeting effector molecules or signaling pathways. miR-223, abundant in resting murine NK cells, is downregulated upon cytokine activation. It targets the 3′ untranslated region (3′UTR) of granzyme B, thereby restraining cytotoxicity in the resting state [45]. Similarly, the miR-15/16 family targets IFN-γ, Bcl2, and cyclin D1, affecting NK cell survival, proliferation, and IFN-γ production—key components of anti-tumor activity [50,51,52].

miR-155 enhances NK cell function by upregulating NKG2D, IFN-γ, and granzyme B. It also modulates SHIP-1, a negative regulator of PI3K-AKT signaling, and impacts T-bet/Tim-3 pathways [21,22]. However, aberrant overexpression of miR-155 in malignant NK cells enhances AKT signaling and may impair surveillance [148]. In contrast, miR-146a, known for its role in dampening inflammation, suppresses NK cell proliferation and cytotoxicity by inhibiting NF-κB signaling [149]. Low miR-146a levels correlate with poor prognosis in extranodal NK/T cell lymphomas.

miR-181a/b promote IFN-γ production by targeting NEMO-like kinase (NLK), while miR-150 regulates NK cell maturation by targeting c-Myb and repressing PI3K-AKT pathway components [17,18]. miR-27a-5p affects NK cell chemotaxis via CX3CR1 regulation and is induced by TGF-β1, which also represses granzyme B and perforin, thereby diminishing NK cytotoxicity [34].

Several tumor-associated miRNAs impair NK cell recognition and function. miR-10b downregulates MICB, a stress ligand for NKG2D, facilitating tumor escape [130]. Similarly, miR-20a, which is upregulated in ovarian cancer, reduces MICA/B expression [54]. In contrast, miR-34a/c, which function as tumor suppressors, upregulate ULBP2 expression, thereby enhancing NK cell-mediated cytotoxicity [43].

miR-29 family members (e.g., miR-29a/b) inhibit melanoma proliferation and modulate the immune checkpoint molecule B7-H3, which suppresses NK cell activity [150]. miR-29b also represses DNA methyltransferases, influencing epigenetic regulation and immune gene expression [58]. miR-30e limits NK cell cytotoxicity by suppressing granzyme B and perforin in CD56^dim^ NK cells [64].

miR-183, which is elevated in renal cell carcinoma, inhibits DAP12 expression—a molecule essential for NK receptor stability—thereby impairing NK cell function [66]. Tumor hypoxia can exacerbate NK cell dysfunction through the TGF-β-dependent upregulation of miR-23a, which is transferred via exosomes [12]. miR-18a, induced by IDO1, downregulates NKG2D and its ligands, reducing NK cytotoxicity. Notably, lncRNA-GAS5 can counteract miR-18a, enhancing IFN-γ secretion and NK cell responses [67].

NK cell trafficking and localization are also miRNA-regulated. miR-27a-5p and miR-561-5p modulate CX3CR1 and CX3CL1, respectively, affecting NK cell infiltration into tumors [34]. In hepatocellular carcinoma, the miR-561-5p/CX3CL1 axis regulates CX3CR1^+^ NK cell recruitment, influencing pulmonary metastasis.

Finally, several miRNAs show therapeutic promise. miR-186, derived from NK cells and exosomes, inhibits neuroblastoma growth by suppressing immune escape mechanisms [68]. In colorectal cancer, miR-146b-5p enhances NK cell activity against chemotherapy-resistant cells by targeting WBSCR22 [91].

### 2.6. Viral miRNAs and NK Cell Evasion

Viral miRNAs also facilitate tumor immune escape. Epstein–Barr virus (EBV)-encoded miR-BART7 downregulates MICA, reducing NK-mediated cytotoxicity in nasopharyngeal carcinoma [69]. Similarly, miR-222 and miR-339, though primarily associated with T cells, downregulate ICAM-1 and may impair NK cell–tumor interactions (Table 1 and Figure 1) [70].

Altogether, miRNAs exert multifaceted control over NK cell development, activation, cytotoxicity, and tumor recognition. Dysregulation of miRNA networks—whether intrinsic to NK cells or driven by tumor-derived factors—can either suppress or enhance NK-mediated immune surveillance. When miRNAs inhibit NK cell function, tumors evade immune detection, leading to progression, immunotherapy resistance, and poor clinical outcomes. Conversely, miRNAs that induce NK cell activity promote immune-mediated tumor clearance and therapeutic responses. A more comprehensive understanding of miRNA-mediated regulation offers promising avenues for therapeutic intervention, including the design of miRNA-based strategies to enhance NK cell function in cancer immunotherapy.

### 2.7. Dendritic Cells (DCs)

Dendritic cells (DCs) are essential antigen-presenting cells that initiate and coordinate anti-tumor immunity. Within the TME, DCs capture tumor-associated antigens (TAAs) and present them to T cells in draining lymph nodes, thereby activating CTLs and promoting adaptive immune responses. Mature DCs are particularly effective at priming CTLs. However, their function is frequently significantly impaired by tumor-derived factors, including miRNAs, which modulate DC maturation, antigen presentation, cytokine secretion, and survival—often promoting immune tolerance and tumor progression. Understanding how miRNAs regulate DCs is vital for improving cancer immunotherapies (Table 1 and Figure 1) [151].

Several miRNAs directly influence DC development and maturation. miR-148a, when inhibited in tumor-associated DCs (TADCs), enhances DC function through the miR-148a/DNMT1/SOCS1 axis. This pathway has been targeted using integrated nano vaccines to boost anti-tumor immunity. In contrast, miR-22 impairs DC anti-tumor function by targeting p38, while miR-146a, upregulated in DCs exposed to pancreatic cancer cells, inhibits maturation and activation by targeting TRAF6 and IRAK1. miR-146a also contributes to endotoxin tolerance, thus acting as a negative regulator of inflammation [71].

miR-155 is crucial for DC development and maturation. Loss of miR-155 impairs responses to infection and immunization, likely through repression of c-Fos as part of a hierarchical regulatory circuit that governs DC activation [20]. Pancreatic cancer-derived exosomes can transfer miR-212-3p to DCs, which inhibits RFXAP—a transcription factor essential for MHC class II expression—thus promoting immune tolerance [74]. Similarly, miR-17-5p, which is upregulated in gastric cancer, suppresses DC maturation and may serve as a biomarker [75].

DC cytokine secretion is modulated by several miRNAs. miR-221 and miR-155 regulate p27^kip1^, KPC1, and SOCS1, which affect IL-12 production, apoptosis, and DC maturation [77]. Let-7i, in response to LPS but no other TLR ligands, suppresses SOCS1 and enhances DC activation. This demonstrates the specificity of miRNA responses in innate immunity [39].

Other miRNAs affect immune checkpoint expression or DC-mediated immune suppression. For example, miR-5119 enhances the immunogenicity of DCs by modulating checkpoint molecules, which improves responses in breast cancer models [78]. By targeting RBP-J and suppressing DC activation, miR-133a acts as a tumor suppressor in osteosarcoma [79]. miR-29b counters the pro-inflammatory, tumor-supporting phenotype of DCs educated by multiple myeloma cells, and it regulates B7-H3, a molecule known to suppress NK cell activity [59].

Tumor-derived exosomal miRNAs also influence DC behavior and immune cell interactions. Microbiota downregulate miR-10a, which in turn targets IL-12/IL-23p40 in DCs and RORA in glioblastoma, altering the differentiation of myeloid-derived suppressor cells (MDSCs) through NF-κB signaling [131]. miR-21, which is found in glioblastoma exosomes, modulates the p-STAT3/p-p65/p-Akt pathway via PTEN targeting, thereby promoting MDSC activation and immune suppression [115].

Moreover, miR-125b regulates inflammation through the type I interferon pathway, and miR-23b promotes tolerogenic DCs by suppressing Notch1/NF-κB signaling [13,14]. These changes reinforce immune suppression in the TME, thereby undermining adaptive immunity.

Additionally, some miRNAs, such as miR-27a-5p and miR-29, though primarily studied in NK cell contexts, also influence DC function indirectly via effects on chemotactic receptors or immunosuppressive ligands. Thus, this highlights the interconnectedness of immune regulatory pathways in the TME.

Taken together, miRNAs are key modulators of dendritic cell development, DC maturation, cytokine secretion, antigen presentation, and immune checkpoint signaling. By regulating these processes, miRNAs dictate the balance between anti-tumor immunity and immune suppression. Tumor-derived miRNAs can reprogram DCs to support immune evasion, while therapeutic modulation of specific miRNAs, such as miR-148a, miR-155, or miR-29b, can restore or enhance DC-mediated immune activation. Understanding these miRNA–DC interactions provides a foundation for developing miRNA-based immunotherapies to improve cancer treatment outcomes by restoring effective antigen presentation and T cell priming.

### 2.8. Tumor-Associated Macrophages (TAMs)

Tumor-associated macrophages (TAMs) are a predominant immune cell population within the TME and play a central role in modulating cancer immune surveillance. Most TAMs exhibit an M2-like, immunosuppressive phenotype characterized by the secretion of anti-inflammatory cytokines and inhibitory molecules that suppress cytotoxic T cell and NK cell activity. In addition to promoting immune evasion, TAMs facilitate tumor progression through enhanced angiogenesis, extracellular matrix remodeling, invasion, and metastasis. Although a subset of TAMs can adopt an M1-like, pro-inflammatory phenotype with anti-tumor properties, TAM accumulation in tumors is generally associated with poor clinical outcomes, underscoring their largely tumor-supportive role [152].

miRNAs are key regulators of TAM biology, influencing their development, polarization, activation, and communication with tumor and immune cells. Several miRNAs promote M1 polarization and pro-inflammatory, anti-tumor responses (Table 1 and Figure 1). For example, miR-125b, miR-27a/b, miR-130a/b, and miR-155 enhance M1 differentiation, leading to increased inflammatory cytokine production and tumoricidal activity. In contrast, miRNAs such as miR-146a, miR-181a, and members of the let-7 family favor M2 polarization, thereby promoting immune suppression and tumor progression [23].

Other than phenotype switching, miRNAs regulate TAM function by modulating intracellular signaling pathways. For example, miR-146a and miR-147 operate within feedback loops that attenuate excessive inflammation by targeting components of the TLR/NF-κB pathway [71,80]. Other miRNAs, such as miR-21 and miR-223, influence TAM–tumor interactions by modulating cytokine secretion and promoting pro-metastatic behaviors [46,47].

Tumor cells can further shape TAM phenotypes via exosomal miRNAs. miR-9, miR-1246, and miR-29b, when transferred from tumor cells to TAMs, skew macrophages toward a tumor-promoting phenotype [60,61,62]. Reciprocally, TAM-derived exosomal miRNAs can affect tumor cells directly. For instance, TAM-secreted miRNAs regulate cancer cell proliferation, drug resistance, and apoptosis through pathways such as PI3K/AKT and PTEN [153].

miRNAs also control TAM metabolism and angiogenic potential. miR-33 regulates macrophage lipid metabolism, which is tightly linked to functional polarization [84]. miR-17 and miR-20a promote angiogenesis by targeting hypoxia-inducible factors (HIFs), which supports vascular remodeling in the TME [55]. Other miRNAs, such as miR-98 and miR-720, modulate TAM-secreted factors that regulate inflammatory cytokine balance and tumor cell migration [85,86].

Interestingly, some miRNAs act as intrinsic brakes on tumor-promoting TAM activity. For example, miR-511-3p, expressed in CD206^+^ TAMs, limits pro-tumor functions, suggesting that endogenous miRNA expression can serve as a regulatory checkpoint that determines macrophage behavior [87].

Together, miRNAs are central regulators of TAM phenotype and function, modulating polarization, signaling pathways, metabolism, angiogenesis, and intercellular communication. Via these mechanisms, miRNAs help determine whether TAMs support or suppress anti-tumor immunity. In many cancers, miRNA dysregulation in TAMs contributes to immune evasion, tumor progression, and therapy resistance. However, specific miRNAs can reprogram TAMs toward an anti-tumor phenotype, highlighting their potential as therapeutic targets. Reprogramming TAMs through miRNA-based strategies represents a promising approach to enhancing cancer immunotherapy and restoring effective tumor immune surveillance.

### 2.9. Tumor-Associated Neutrophils (TANs)

Tumor-associated neutrophils (TANs) play a complex, dual role in cancer immune surveillance. During early tumor development, TANs can promote anti-tumor immunity by presenting antigens and secreting pro-inflammatory cytokines. However, as tumors progress, TANs frequently adopt a pro-tumorigenic phenotype. As N2-polarized TANs, they suppress CD4^+^ and CD8^+^ T cell activity through mechanisms such as myeloperoxidase (MPO) and Fas/FasL expression, while they also release immunosuppressive factors that promote tumor growth, metastasis, and immune evasion. The dynamic polarization of TANs between anti-tumor (N1) and pro-tumor (N2) states is regulated by signals in the TME, including miRNAs, which orchestrate neutrophil development, activation, and function (Table 1 and Figure 1) [154].

Several miRNAs regulate neutrophil extracellular trap formation (NETosis), a form of neutrophil cell death, and influence TAN inflammatory activity. miR-155 promotes NETosis by upregulating PAD4, enhancing trap formation with implications for both antimicrobial defense and tumor-associated thrombosis. Knockdown of miR-155-5p reduces PAD4 mRNA and NET generation [24]. miR-146a also regulates NETosis by targeting the TLR4/NF-κB pathway. In infectious and inflammatory settings, it enhances IL-8 and CCL5 production, linking it to tumor-associated inflammation [48]. miR-1696, another promoter of NETosis, represses GPx3, suggesting roles in oxidative stress and immune cell recruitment within the TME [93]. Other NETosis-associated miRNAs include miR-7, miR-223, miR-3146, and miR-16-5p, which are being explored as therapeutic targets for NET suppression. Notably, miR-223 also regulates myeloid precursor proliferation via IGF-R signaling, influencing neutrophil maturation and recruitment. miR-16-5p, in addition to regulating NETosis, modulates autophagy, a key determinant of neutrophil survival and function in tumors [48,49].

Differential miRNA expression profiles help define TAN phenotypes. In colon cancer, miR-4780 is upregulated and targets TUSC1, while miR-3938 is downregulated and targets ZNF197, suggesting that these miRNAs promote N2 pro-tumorigenic characteristics [94]. Although the regulation of N1/N2 polarization by miRNAs remains underexplored, such patterns provide insight into subtype-specific functions and therapeutic vulnerabilities.

Other miRNAs also contribute to TAN specialization. miR-138, although primarily studied in tumor cells, suppresses neutrophil-derived NGAL (lipocalin 2), reducing tumor proliferation and metastasis in pancreatic cancer models [95]. let-7b exerts anti-inflammatory effects in neutrophils by downregulating TLR4, IL-6, IL-8, and TNF-α, while upregulating IL-10, thus modulating inflammatory signaling and neutrophil recruitment [97].

TAN recruitment and trafficking are also regulated by miRNAs. miR-17 and miR-31 promote neutrophil adhesion and migration by upregulating IL-8 [17]. miR-130a controls neutrophil maturation via MPO, proteinase 3, TGF-β1, and controls cell cycle exit [35]. miR-199 and miR-722 inhibit neutrophil chemotaxis and TAN infiltration to potentially limit immune surveillance [100,101]. miR-21, a well-established regulator of inflammation, also acts in TANs to shape the cytokine milieu and immunosuppressive tone of the TME [155].

Cross-talk with other immune cells is also modulated by miRNAs. miR-142-3p regulates neutrophil–macrophage interactions by modulating TNF-α production and inhibiting PKC-α in macrophages [38,102]. Given the cooperative interactions between neutrophils and macrophages in the TME, this miRNA likely contributes to the broader immune regulatory landscape.

The heterogeneity of TANs includes immunosuppressive immature cells, often classified as polymorphonuclear myeloid-derived suppressor cells (PMN-MDSCs), which suppress T cells via reactive oxygen species (ROS) and arginase-1. miR-125a-5p may contribute to the development and suppressive function of PMN-MDSCs, although direct evidence remains limited [103].

Several miRNAs—miR-155, miR-146a, miR-1696, and miR-223—are promising therapeutic targets for NETosis suppression, a strategy that may reduce thrombosis and reprogram the TME toward immune activation [25,26,27]. Targeting neutrophil-associated miRNAs thus represents a novel approach to modulate TAN function, control tumor progression, and enhance anti-tumor immunity.

Taken together, miRNAs act as central regulators of TAN behavior, influencing their development, recruitment, activation, polarization, and survival within the TME. Through modulation of NETosis, cytokine production, chemotaxis, and cross-talk with other immune or stromal cells, miRNAs shape the functional plasticity of TANs and influence whether they support or suppress anti-tumor immunity. Their differential expression in N1 versus N2 TANs and their responsiveness to tumor-derived signals highlight their role in neutrophil plasticity and functional heterogeneity. As key molecular switches, miRNAs offer potential as biomarkers of TAN activity and as therapeutic targets for reprogramming neutrophils to favor anti-tumor immune responses.

## 3. miRNAs and Immune Equilibrium

Tumor immune equilibrium represents a critical transitional phase in cancer immunoediting, where the immune system and tumor coexist in a dynamic, prolonged stalemate. Unlike the elimination phase of immune surveillance, immune equilibrium involves partial immune control, which is sufficient to prevent overt tumor progression but insufficient to completely eradicate tumor cells. This phase can persist for months to years and is shaped by both immunostimulatory and immunosuppressive mechanisms within the TME (Table 1 and Figure 1).

### 3.1. T Cells

Cytotoxic T lymphocytes are key effectors that can eliminate tumor cells, but in equilibrium, they often become partially dysfunctional or “exhausted,” expressing inhibitory checkpoints and producing fewer cytokines. miRNAs play dual roles in either sustaining T cell anti-tumor activity or promoting T cell exhaustion, thereby modulating the equilibrium (Table 1 and Figure 1). For example, miR-31 is induced upon T cell receptor activation and promotes CD8^+^ exhaustion [98,99]. miR-31 increases the transcription factor c-Maf and the PGE₂ receptor Ptger2, which in turn upregulate inhibitory checkpoint receptors PD-1 and LAG3, and the immunosuppressive cytokine IL-10. This leads to reduced expression of cytotoxic effector molecules, such as perforin and granzymes, in T cells, thereby curbing their tumor-killing ability. Another example is miR-24. Found in hypoxic TMEs, e.g., in nasopharyngeal carcinoma, this miRNA drives activated T cells into exhaustion by upregulating the immunosuppressive ectoenzyme CD39, and the checkpoint molecules PD-1 and TIM-3. It also dampens T cell metabolism by targeting Myc and FGF11, reducing mitochondrial function [36]. These miRNAs help tumors induce T cell dysfunction, thereby maintaining an equilibrium where T cells cannot fully eradicate the cancer.

Conversely, some miRNAs support T cell activity and persistence, preventing immune elimination from tipping into escape. miR-155 is a context-dependent example. In acute immune responses, miR-155 is a well-known booster of T cell effector functions, among others, by targeting SOCS1 to enhance cytokine signaling [156,157]. In tumors, miR-155 appears to preserve T cells in a less exhausted state. In a melanoma model, miR-155 was upregulated in tumor-infiltrating CD8^+^, and knockout of miR-155 led to accelerated tumor growth with more exhausted T cells. miR-155 deficiency impaired T cell cytokine production via the SOCS-1/STAT5 pathway, resulting in higher expression of PD-1 and other checkpoint molecules—hallmarks of T cell exhaustion [158]. Thus, miR-155 can inhibit T cell exhaustion, supporting a prolonged immune response during equilibrium. Similarly, miR-149-3p was identified as a “reinvigorating” miRNA in T cells. In a breast cancer model, miR-149-3p was enriched in less-exhausted (PD-1) CD8^+^ and its overexpression enhanced T cell proliferation and IFN-γ/TNF-α production [28]. Mechanistically, miR-149-3p directly targets multiple checkpoint receptors, e.g., PD-1, TIM-3, BTLA, and the transcriptional repressor Foxp1, thereby reversing exhaustion and restoring T cell cytotoxicity. Thus, these miRNAs help immune cells contain the tumor, preventing complete escape.

Other miRNAs fine-tune T cell responses at the brink of equilibrium. For instance, miR-28 is downregulated in exhausted CD8^+^ in melanoma. Restoration of miR-28 directly suppresses the checkpoint molecules PD-1, TIM-3, and BTLA [106], resulting in a revival of T cell activity. In contrast, miR-15a/16, which is a tumor-suppressive cluster in other contexts, can restrain T cell function. Indeed, knockout of miR-15a/16 in a glioma model led to more robust CD8^+^ responses and tumor control [53]. The miR-15a/16 gene cluster normally targets mTOR. However, when these miRNAs are absent, T cells enhance proliferation and cytokine secretion with lower expression of the PD-1, TIM-3, and LAG-3 checkpoint receptors. This suggests that in the tumor milieu, high miR-15a/16 may contribute to T cell hyporesponsiveness, whereas their loss tips the balance toward elimination. Similarly, in lung cancer, tumor-derived circular RNA circ_002178 sponges miR-34, leading to increased tumor PD-L1 and also shuttling into CD8^+^ to upregulate PD-1 [159]. This miR sequestration blunts T cell activity, exemplifying how tumors exploit miRNA networks to enforce equilibrium.

Notably, across cancers, a pattern emerges that miRNAs such as miR-31, miR-24, and miR-15a/16 favor a restrained T cell state, which prevents full tumor elimination, whereas miR-155, miR-149-3p, and miR-28 bolster T cell activity to keep tumors in check without entirely escaping. For example, in breast cancer, miR-424-5p and miR-138-5p were found downregulated in TILs. Yet, restoring their expression decreased tumor cell viability and T cell exhaustion by targeting tumor PD-L1, thereby reducing IL-10 and boosting IL-2/IFN-γ [123,124]. In hepatocellular carcinoma (HCC), HBV-driven tumors suppress miR-200c via TGF-β/STAT3 signaling, which in turn elevates PD-L1, while reintroduction of miR-200c reduces PD-L1 expression and reinvigorates T cells [107]. These examples illustrate how miRNAs orchestrate the delicate equilibrium between T cell attack and tumor survival.

### 3.2. Regulatory T Cell (Treg)

FoxP3^+^ Tregs accumulate in many tumors and suppress anti-tumor immunity, thereby supporting immune equilibrium by preventing complete immune elimination. miRNAs influence both Treg abundance and suppressive function in the TME.

One strategy to disrupt tumor-protective Tregs is via miRNAs that impair Treg survival or function (Table 1 and Figure 1). miR-125b-5p is a notable example that directly targets the Treg lineage. Overexpression of miR-125b-5p was shown to inhibit Treg proliferation and suppressive activity by downregulating TNFR2 and FoxP3 [160]. In a colorectal cancer model, delivering miR-125b-5p via MSC-derived exosomes curtailed intratumoral Treg expansion and function, leading to enhanced CD8^+^ responses and tumor regression. Treated mice saw significantly reduced tumor growth, especially when miR-125b-5p therapy was combined with anti-PD-1 checkpoint blockade. This demonstrates that miR-125b-5p can tilt the equilibrium by weakening Treg-mediated immunosuppression, allowing effector T cells to better control the tumor.

miRNAs can also act in tumor or other stromal cells to influence Treg recruitment. miR-34a is a tumor-suppressor miRNA often silenced by TGF-β in cancer cells [42]. In HBV-related HCC, loss of miR-34a leads to increased production of the chemokine CCL22, which recruits Tregs into tumors [44]. An inverse correlation is observed between miR-34a and Treg levels (FoxP3^+^ cells) in patient tumors. Restoration of miR-34a in HCC models reduced CCL22 secretion, thereby reducing Treg infiltration and resulting in slowed tumor growth and metastasis. In the context of immune equilibrium, tumor-downregulation of miR-34a promotes Treg recruitment and thereby enhances local immunosuppression, whereas restoration of miR-34a shifts the balance toward immune control by limiting Treg recruitment.

Intrinsic miRNAs in Tregs can modulate their immune suppressive function in the TME. In melanoma, Tregs show lower CTLA-4 levels than normal, a phenomenon linked to miR-155. Tumor-derived factors induce high miR-155 in Tregs, which post-transcriptionally silences CTLA-4 mRNA [161]. Since CTLA-4 on Tregs suppresses immunity by sequestering costimulatory ligands on antigen-presenting cells, miR-155-mediated CTLA-4 downregulation can paradoxically reduce Treg suppressive capacity. This was associated with worse outcomes in metastatic melanoma, as low Treg CTLA-4 might reflect a state of Treg dysfunction or adaptation in equilibrium. Targeting this pathway, for example, by inhibiting miR-155 in Tregs, could restore Treg CTLA-4 and potentially alter the balance in favor of anti-tumor immunity. Another miRNA, miR-146a, known to stabilize Treg identity in inflammatory settings, may also play a role in tumors. High miR-146a in Tregs suppresses pro-inflammatory signaling, thereby stabilizing their suppressive phenotype [162]. Although direct tumor-specific data are sparse, one can infer that a loss of miR-146a in tumor Tregs may destabilize them and reduce immunosuppression, whereas high miR-146a helps maintain the equilibrium by keeping Tregs functional.

Taken together, miRNAs can either diminish Treg-mediated immunosuppression (e.g., miR-125b-5p and miR-34a) or fine-tune Treg function (e.g., miR-155 and miR-146a), thereby impacting the level of immune suppression in the TME. By controlling Treg abundance and activity, these miRNAs determine whether the tumor is held in a dormant immune equilibrium or progresses towards escape.

### 3.3. Tumor-Associated Macrophages (TAMs)

In the TME, macrophages often polarize toward either pro-inflammatory M1 phenotypes (tumoricidal, immune-stimulating) or anti-inflammatory M2 phenotypes (tumor-promoting, immunosuppressive). The balance between M1 and M2 TAMs is pivotal in tumor immune equilibrium. miRNAs are key regulators of this polarization, as they influence whether macrophages support immune activation or suppression (Table 1 and Figure 1).

miR-21 is one of the most abundant oncogenic miRNAs (oncomiRs) in tumors and is highly expressed in macrophages [163,164]. In TAMs, miR-21 promotes immunosuppressive, pro-tumoral functions. Loss of miR-21 dramatically shifts macrophages toward an M1-like state. Sahraei et al. showed that miR-21 deletion in TAMs skewed their transcriptome to a pro-inflammatory, angiostatic phenotype and significantly suppressed tumor growth. miR-21-deficient TAMs produced higher levels of IL-12 and CXCL10—a potent Th1 cytokine and T cell chemoattractant—leading to enhanced CD8^+^ activity in the TME. Tumors in miR-21^–/–^ mice showed reduced neovascularization and increased tumor cell death. Thus, miR-21 normally promotes an M2, immunosuppressive TAM program, helping tumors evade immunity. High miR-21 in TAMs, as observed in lung, breast, and other cancers, supports the equilibrium by restraining macrophage anti-tumor activity and aiding tumor survival. In contrast, miR-21 inhibition unleashes macrophage-driven anti-tumor immunity.

Unlike miR-21, miR-155 is a prototypical inflammatory miRNA that drives M1 polarization. It is elevated in classically activated macrophages and targets negative regulators of inflammation, such as C/EBPβ and SOCS1 [23]. Overexpression of miR-155 reprograms M2-polarized TAMs toward an M1 state, increasing their IL-12 production and tumoricidal activity [165]. Conversely, silencing miR-155 skews macrophages to M2 and accelerates tumor growth. In a mammary tumor model, the lack of miR-155 led to more immunosuppressive TAMs and consequently faster tumor progression. Notably, miR-155 has multifaceted roles. While it strengthens T cell and macrophage responses, it also supports the generation of myeloid-derived suppressor cells and Tregs in some contexts. However, within TAMs specifically, miR-155’s net effect promotes an M1 phenotype, via high IL-12 and TNF-α, and inhibits the M2 program [166]. Thus, miR-155 in TAMs counteracts tumor immune evasion. Its presence supports immune elimination, and its absence facilitates equilibrium or escape by allowing M2 dominance.

miR-146a is an anti-inflammatory miRNA induced by NF-κB as part of a negative feedback loop. It is expressed at higher levels in M2-polarized TAMs than in M1 macrophages [23]. miR-146a directly targets signaling adapters such as TRAF6 and IRAK1 downstream of Toll-like receptors, thereby dampening NF-κB activation and inflammatory cytokine production. This activity helps maintain the immunosuppressive, tumor-promoting phenotype of TAMs. In fact, inhibition of miR-146a in TAMs was shown to decrease M2 gene expression and impair tumor growth. Suppressing IL-12 and other M1-associated cytokines while promoting IL-10, TGF-β, and miR-146a allows macrophages to support tumor immune evasion. Other miRNAs enriched in M2 TAMs, such as miR-125a and miR-145-5p, further reinforce macrophage-mediated suppression. Interestingly, some miRNAs expressed in M2 TAMs counterbalance excessive polarization. For example, miR-511-3p, which is co-transcribed with the M2 marker gene *CD206*/*MRC1*, paradoxically inhibits M2-associated gene expression when it is overexpressed, indicating a complex regulatory network [23,88,89].

Overall, miRNAs act as molecular switches between tumoricidal and tumor-protective macrophage states, thereby governing the equilibrium. Targeting these miRNAs may tip the balance—for example, inhibiting miR-21 can promote anti-tumor immunity, whereas delivering miR-146a mimics may support immunosuppression and tumor dormancy.

### 3.4. Dendritic Cells (DCs)

DCs orchestrate anti-tumor T cell responses by processing and presenting tumor antigens and providing co-stimulation. In tumor immune equilibrium, DCs are often partially tolerized, i.e., present but with impaired capacity to fully activate T cells.

Tumors can secrete miRNA-loaded exosomes that condition DCs toward a tolerogenic state (Table 1 and Figure 1). A striking example is pancreatic cancer, where tumor-derived exosomes deliver miR-212-3p to DC. Ding et al. found that miR-212-3p taken up by DCs targets RFXAP, a transcription factor essential for MHC class II expression [74]. As a result, MHC II expression is downregulated in DCs exposed to pancreatic cancer exosomes. With reduced antigen presentation capacity, these DCs cannot effectively prime CD4^+^ T cells, contributing to an immune-tolerant milieu. Clinically, pancreatic cancer patient samples showed an inverse correlation between miR-212-3p and RFXAP levels. By suppressing MHC II antigen presentation, tumor-secreted miR-212-3p helps maintain equilibrium; the immune system is not fully alerted to the tumor’s presence, thus preventing outright elimination.

Intrinsic miRNAs in DCs modulate their production of cytokines like IL-12, which promotes Th1 and CTL responses, versus IL-10, which induces tolerance. miR-146a and miR-146b are notable in human monocyte-derived DCs, as they inhibit the TLR/NF-κB pathway by targeting TRAF6 and IRAK1. In turn, this leads to lower IL-12p70, IL-6, and TNF-α production [92]. Upregulation of miR-146a/b in tumor-infiltrating DCs would thus dampen pro-inflammatory cytokines and possibly promote DC apoptosis or exhaustion, as suggested by in vitro studies. Indeed, miR-146a is often induced by the IL-10-rich tumor environment and could enforce a “brake” on DC activation, supporting a state of immune equilibrium where T cells receive suboptimal stimulation. In contrast, miR-155 is a positive regulator of DC immunogenicity. miR-155-deficient DCs show defective antigen presentation and cytokine secretion [20]. In tumors, miR-155 levels in DCs may be suppressed by factors like TGF-β. This would limit DCs in their ability to stimulate T cells, thus contributing to the equilibrium. Conversely, augmenting miR-155 in DCs can enhance their IL-12 output and T cell priming capacity, which may break the equilibrium toward elimination. miRNAs act as modulators of DC function in the TME, often tipping them toward a semi-mature, low IL-12 state that supports tumor persistence. By limiting antigen presentation (e.g., via miR-212-3p) and inflammatory cytokine release (e.g., via miR-146a), these miRNAs ensure that the anti-tumor immune response remains muted—sufficient to constrain tumor growth, but insufficient to achieve eradication.

### 3.5. Natural Killer (NK) Cells

in the equilibrium phase, NK cells often become functionally impaired by the TME, showing features sometimes described as NK cell “exhaustion” or anergy (Table 1 and Figure 1).

TGF-β is abundant in many TMEs and is a potent inhibitor of NK cell activity. A pivotal discovery was that TGF-β mediates NK cell suppression in part through miRNAs. miR-183 is strongly upregulated in NK cells upon TGF-β exposure. Donatelli et al. showed that TGF-β-induced miR-183 directly targets DAP12 (TYROBP), the adaptor protein required for signaling by several activating NK receptors [66]. Loss of DAP12 leads to downregulation of surface NK receptors such as NKG2C, NKp44, and the activating KIRs, impairing NK cytotoxic function. In lung cancer patients, tumor-infiltrating NK cells exhibited reduced DAP12 expression, consistent with this miR-183-mediated pathway. Thus, TGF-β, via miR-183, enforces NK-cell silencing, helping tumors to avoid NK surveillance and maintaining equilibrium. Another TGF-β-responsive miRNA, miR-1245, has been shown to target NKG2D, a key activating receptor on NK cells. In the TME, high miR-1245 expression causes NKG2D downregulation, thereby reducing NK recognition of tumor ligands and impairing NK cytotoxicity [167,168]. In melanoma and other cancers, TGF-β and associated miRNAs such as miR-183 and miR-1245 create an NK cell phenotype that can no longer effectively attack tumor cells, contributing to the immune equilibrium where NK cells are present but not fully functional.

Tumors also modulate NK cells via other pathways. miR-30e, miR-27a, and miR-378 have been identified as inhibitory miRNAs in NK cells that are elevated under chronic IFN or IL-15 stimulation and can reduce granzyme B and perforin levels [64,65]. For instance, miR-30e was shown to suppress translation of *PRF1*, encoding perforin, and impair NK killing of tumor targets. In contrast, IL-12/IL-15-indiced miR-155 is a positive regulator in NK cells, enhancing their IFN-γ production and cytotoxicity by targeting the NK inhibitor SHIP-1 [169,170]. In the TME, pro-inflammatory cytokines may induce miR-155 expression in NK cells to sustain some level of activity. However, many tumors exhibit dominant suppressive signals. For example, in non-small cell lung cancer (NSCLC) patients, NK cells were found to have notably reduced miR-130a levels and concomitantly elevated STAT3 activity [171]. STAT3 is a transcription factor that, when active in NK cells, dampens their cytotoxic program. Indeed, in NK cells, restoring miR-130a, which targets STAT3, enhanced NK cytotoxicity against lung cancer cells. This suggests that tumors downregulate miR-130a to unleash inhibitory effects of STAT3, keeping NK cells in a subdued state.

Notably, NK cells can also secrete miRNAs in exosomes that impact tumor cells. For instance, NK exosomes enriched in let-7b can be delivered to tumor cells to suppress their proliferation [172]. During equilibrium, this bidirectional exchange can help contain tumor growth. However, more commonly, tumor-derived factors dominate, and these cause NK cells to express inhibitory miRNA profiles. In the equilibrium state of melanoma, lung, and other cancers, NK cells often express immune checkpoint molecules like PD-1 and show altered receptor repertoires [168]. miRNAs are interwoven in these processes. Key examples are miR-138 and miR-424, which modulate PD-L1/PD-1 signaling in tumor and immune cells, indirectly affecting NK cell interactions with tumors [96].

Thus, in NK cells, miRNAs serve as intracellular master regulators that can either arm NK cells to fight (e.g., miR-155 and miR-130a) or disarm them (e.g., miR-183 and miR-1245). The tumor milieu tends to induce the latter, imposing an equilibrium where NK cells are restrained. Reversing these miRNA changes may overcome inhibitory programming and re-activate NK cell cytotoxicity to tip the balance toward tumor clearance.

## 4. miRNAs and Immune Evasion

miRNAs are key modulators of tumor immune evasion, the process in which cancer cells avoid detection and destruction by the immune system. This evasion is a major challenge in cancer treatment, particularly in immunotherapy [173] (Table 1 and Figure 1).

### 4.1. Regulatory T Cells (Tregs)

Tregs play a critical role in maintaining immune tolerance and suppressing anti-tumor immunity within the TME. Several miRNAs regulate Treg differentiation, expansion, and immune suppressive functions, significantly contributing to tumor immune evasion and progression [174].

Tumor-derived miRNAs often promote Treg expansion or stability (Table 1 and Figure 1). For example, miR-214 is secreted by tumor cells and delivered to Tregs via microvesicles, where it downregulates PTEN to promote Treg expansion and secretion of IL-10, enhances immune suppression and tumor growth [41]. Similarly, miR-192-5p enhances Treg differentiation through the RB1/NF-κBp65 signaling axis, reinforcing IL-10 secretion and immune suppression [40], while miR-4772-3p upregulates Helios (IKZF2), promoting Treg proliferation in NSCLC [108].

Several miRNAs facilitate Treg recruitment to tumors. miR-141 promotes the recruitment of Tregs to the tumor site via CXCL1 production, which aids NSCLCs in immune evasion. Similarly, miR-128-3p influences Treg enrichment in gastric cancer by interacting with IL16 to promote immune evasion [109], and miR-22 activates JAK/STAT3 signaling, which leads to the secretion of chemokines such as CCL17, CCL20, and CCL22, recruiting Tregs to the TME and establishing an immunosuppressive environment [73].

Other miRNAs directly modulate Treg suppressive activity. In breast cancers, miR-182 targets FOXO1 and NFATs, increasing FOXP3 and TGF-β expression, which promotes Treg polarization and the formation of IL-17-producing Tregs, facilitating immune evasion [110]. In NSCLC, miR-17-5p suppresses RUNX3, promotes Treg-mediated immune suppression [76]. In OSCC, miR-325-3p stabilizes Foxp3, which is crucial for Treg function [111].

Other tumor-derived miRNAs include miR-208b, which in colorectal cancer promotes Treg expansion and chemotherapy resistance by targeting PDCD4 [112]. Likewise, miR-423-5p interacts with FENDRR to support Treg-mediated immune evasion in HCC [113], while finally, miR-155-5p and miR-194-5p enhance Treg function, the latter via PD-L1 modulation [29]. Interestingly, miR-429 targets PD-L1 to reduce Treg infiltration in HCC, suggesting a context-dependent, potentially anti-immunosuppressive role [114].

Collectively, these miRNAs shape Treg differentiation, expansion, and function, which reinforces an immunosuppressive TME that promotes tumor progression. Understanding how these miRNAs interact provides valuable insights with the potential to develop new therapeutic strategies to target Tregs and enhance anti-tumor immunity.

### 4.2. Myeloid-Derived Suppressor Cells (MDSCs)

MDSCs are immunosuppressive cells within the TME. They inhibit both innate and adaptive immune responses by preventing T cell activation and promoting the expansion of other immunosuppressive cell populations [175,176,177]. This contributes significantly to tumor growth, metastasis, angiogenesis, resistance, and poor clinical outcomes. A variety of miRNAs regulate MDSC differentiation, activation, and function, thus contributing to immune evasion and tumor progression (Table 1 and Figure 1) [178,179].

Hypoxia-associated miRNAs are especially important for promoting MDSC-mediated immunosuppression in glioma. Hypoxic stress induces expression of miR-10a, miR-21, and miR-210. This promotes the expansion and activation of MDSCs, as well as T cell inhibition [115,116]. Another important miRNA, miR-1246, is abundant in exosomes from glioma cells and promotes the differentiation and activation of MDSCs through the DUSP3/ERK-dependent pathway, which further enhances MDSC-mediated suppression of immune responses [83]. Similarly, miR-29a and miR-92a, discovered in glioma-derived exosomes, enhance MDSC differentiation and function by targeting Hbp1 and Prkar1a. This action contributes to the establishment of an immunosuppressive environment [56].

Other tumor-derived miRNAs, including miR-146a, miR-155, miR-125b, miR-100, let-7e, miR-125a, miR-146b, and miR-99b, have been shown to drive the conversion of monocytes into MDSCs, particularly in melanoma. This supports immune evasion and resistance to immunotherapy [90]. However, while miR-1298-5p has been shown to inhibit glioma progression, paradoxically it enhances the immunosuppressive effects of MDSCs. This highlights the context-dependent complexity of miRNA activity in tumor immunity [117].

In breast cancers, miR-9 and miR-181a from exosomes contribute to the early development of MDSCs by targeting SOCS3 and PIAS3, key regulators of the JAK/STAT pathway. This disruption enhances MDSC differentiation, boosting immune suppression and aiding tumor progression [57]. In gastric cancer, miR-494, regulated by TGF-β, is upregulated and potentially reduces the immunosuppressive effects of MDSCs. While its effect seems to counteract MDSC-mediated suppression, its role remains complex, and more research is needed to fully understand its impact on tumor immunity [119].

Together, these miRNAs orchestrate MDSC recruitment, differentiation, and suppressive functions, thereby reinforcing an immunosuppressive TME in glioma, melanoma, and probably other types of cancer. Targeting miRNAs that regulate MDSCs may constitute new strategies to advance the efficacy of immunotherapies.

### 4.3. Tumor-Associated Macrophages (TAMs)

Tumor-associated macrophages (TAMs) are crucial regulators of immune evasion and tumor progression. They modulate T cell activity and immune checkpoint expression and secrete immunosuppressive cytokines. Together, this leads to the formation of immune-privileged sites in which tumor cells are protected from the immune system [180,181,182]. Additionally, TAMs play a key role in promoting tumor angiogenesis and metastasis, which further promotes immune evasion and cancer spread [183].

Several tumor-derived miRNAs influence TAM behavior to favor immune suppression (Table 1 and Figure 1). For example, in breast cancer, miR-375 is released from apoptotic tumor cells and taken up by TAMs via the CD36 receptor. This miRNA enhances macrophage migration and infiltration into tumors, promoting a tumor-supportive microenvironment. It targets TNS3 and PXN in macrophages, while also regulating CCL2 expression in tumor cells, creating a supportive niche for tumor progression [120]. In pancreatic cancer, miR-155-5p is derived from tumor cells and taken up by TAMs to promote M2 polarization via Akt/NF-κB signaling, thereby fostering an immunosuppressive TME [184]. Similarly, miR-125b-5p, originating from melanoma exosomes, is taken up by TAMs to induce a tumor-promoting phenotype through the targeting of lysosomal acid lipase A (LIPA) [105].

Other miRNAs influence macrophage polarization. Modulated by Notch signaling, miR-125a redirects TAMs towards an M1-like phenotype to enhance their anti-tumor efficacy [104]. In contrast, in both breast and ovarian cancer, miR-101 facilitates the transition from M1 to M2 macrophages, enhancing cancer cell proliferation and migration. This miRNA modulates the expression of C/EBPα and KLF6, contributing to tumor progression [121]. These examples highlight the therapeutic potential of manipulating macrophage phenotypes for cancer treatment.

TAM-derived exosomal miRNAs can also modulate other immune cells in the TME. miR-29a-3p and miR-21-5p, found in exosomes from TAMs, can create an imbalance between Treg and Th17 cells by specifically targeting STAT3 in CD4^+^ T lymphocytes. This establishes an immunosuppressive TME that promotes tumor progression and metastasis, particularly in epithelial ovarian cancer [57]. This is similar for miR-21, which is elevated in TAMs. Inhibiting miR-21 in TAMs can drive a transition to a pro-inflammatory phenotype, which boosts anti-tumor immune responses and enhances the efficacy of immune therapies [163,185].

miR-100 is another key miRNA present in TAMs, which sustains their pro-tumor characteristics by influencing the mTOR pathway. It is linked to the Stat5a/IL-1ra pathway, which promotes tumor metastasis in a mouse model of breast cancer [122].

Together, these miRNAs regulate TAM polarization, migration, and intercellular communication, thereby promoting tumor immune evasion, progression, and metastasis. Understanding the molecular pathways controlled by these miRNAs may open up new possibilities for therapeutic strategies aimed at modulating TAM behavior to enhance anti-tumor immunity and improve treatment outcomes.

### 4.4. Cancer-Associated Fibroblast (CAF)

Cancer-associated fibroblasts (CAFs) play a significant role in the TME by releasing exosomal miRNAs that regulate various aspects of tumor biology, including immune modulation (Table 1 and Figure 1). These miRNAs promote immune evasion, tumor growth, and resistance to therapy, making them crucial mediators of cancer progression [186]. In esophageal squamous cell carcinoma, miR-21 is released by CAFs in exosomes, where, together with IL-6, it stimulates MDSC generation through STAT3 signaling. This contributes to immune evasion and increases drug resistance [81]. Similarly, in gastric cancer, miR-149 is epigenetically silenced in CAFs, enhancing IL-6 secretion. This miRNA also links prostaglandin E2 (PGE2) and IL-6 signaling, promoting epithelial–mesenchymal transition (EMT) and stem-like properties in cancer cells, which aids in immune evasion and tumor progression [125].

In NSCLC, miR-20a is delivered via CAF-derived exosomes, where it suppresses the PTEN/PI3K-AKT pathway. This action enhances cell proliferation, chemoresistance, and immune evasion, thus promoting tumor progression [187]. Additionally, in breast cancer, CAF-produced miR-181d-5p is taken up by cancer cells via exosomes to inhibit CDX2 and HOXA5, which promotes EMT and increases tumor aggressiveness. This miRNA boosts tumor cell proliferation and invasion, further aiding immune evasion [118]. Also, in breast cancer, CAF-produced exosomes containing miR-500a-5p are taken up by cancer cells to target USP28 and facilitate proliferation, metastasis, immune evasion, and dissemination [126]. Conversely, in bladder cancer, miR-148b-3p is reduced in CAF-derived exosomes, which enhances chemosensitivity by suppressing the Wnt/β-catenin pathway and elevating PTEN expression [72]. Additionally, in lung squamous cell carcinoma, CAF-derived miR-369 stimulates the MAPK signaling pathway, enhancing cancer cell migration and invasion, thus potentially facilitating immune evasion [127]. Finally, miR-196a, found at elevated levels in CAFs, promotes the migration and invasion of lung cancer cells by targeting ANXA1 and enhancing CCL2 secretion. This miRNA influences immune cell recruitment and function, further supporting immune evasion and metastasis [128].

Thus, together, these CAF-derived miRNAs establish an immunosuppressive TME by modulating immune cell recruitment, inflammatory signaling, and tumor–stroma interactions. In turn, this promotes immune evasion, therapy resistance, and cancer progression.

### 4.5. Endothelial Cells of the Tumor Vasculature

Angiogenesis, i.e., blood vessel formation, and the endothelial cells of the tumor vascular are crucial for tumor evasion, as they support tumor growth and immune escape. Tumors exploit angiogenesis to create a non-adhesive vasculature that prevents immune cell infiltration, a phenomenon known as tumor endothelial cell anergy. This is induced by angiogenic growth factors, which allow tumors to mimic embryonic conditions and create an immune-privileged environment that promotes tumor progression [188].

Several miRNAs play significant roles in endothelial cell dysfunction and tumor angiogenesis (Table 1 and Figure 1). The miR-181 family members regulate endothelial cell behavior, suggesting that targeting them could be a potential strategy for anti-angiogenic therapies in tumor treatment [189]. In response to VEGF stimulation, miR-10b and miR-196b are upregulated in the vasculature of high-grade breast tumors. Targeting these miRNAs significantly reduced angiogenesis and tumor growth in mice. This demonstrates their pivotal role in tumor angiogenesis and their potential as therapeutic targets [129]. In epithelial ovarian cancer, miR-141-3p is released in extracellular vesicles from cancer cells. It enhances angiogenesis in endothelial cells by activating critical signaling pathways, including JAK/STAT3 and NF-κB. This promotes endothelial cell migration and angiogenesis, supporting tumor growth and immune evasion, making miR-141-3p a key player in tumor vascularization [132].

Several pro-angiogenic miRNAs exist, such as miR-1468-5p, which is secreted by cervical cancer cells in exosomes. This facilitates immune evasion by enhancing PD-L1 expression and promoting lymphangiogenesis in lymphatic endothelial cells (LECs). This miRNA triggers the JAK2/STAT3 pathway, fostering an immunosuppressive environment that weakens T cell immunity, further contributing to tumor immune escape [133]. Furthermore, miR-526b and miR-655, induced by COX-2, significantly contribute to angiogenesis and lymphangiogenesis in breast cancer. They facilitate this by upregulating VEGF markers and downregulating the tumor suppressor PTEN, activating the PI3K/Akt and EP4 signaling pathways, which are critical for angiogenesis and tumor progression [134]. In addition, miR-9 and miR-494 are overexpressed in tumors and taken up by endothelial cells, where they contribute to endothelial cell migration and angiogenesis, thus promoting tumor growth and immune evasion [82].

In contrast, several anti-angiogenic miRNAs suppress tumor vascularization. For example, miR-29b inhibits angiogenesis and tumor growth in breast cancer. It suppresses angiogenesis by targeting Akt3 and inhibiting VEGF and C-myc expression. This suggests that miR-29b could serve as a promising anti-cancer therapeutic agent by inhibiting tumor vascularization and progression [63]. Similarly, miR-200 and miR-128 are downregulated in tumors and inhibit migration and angiogenesis [82]. miR-103 controls tumor-associated endothelial cells by enhancing tumor cell death. It exacerbates DNA damage and inhibits angiogenesis, making miR-103 a potential therapeutic candidate for disrupting tumor vascularization and promoting tumor regression [135].

Thus, jointly, these miRNAs regulate tumor vascularization, immune cell access, and angiogenic signaling. As such, they modulate tumor progression and immune escape. Targeting endothelial cell-associated miRNAs represents a promising strategy for both anti-angiogenic and immunotherapeutic treatments.

## 5. miRNAs and Immunometabolism

Immunometabolism is a vital field that examines how metabolic processes within immune cells are functionally connected to immune responses, including cell activation, proliferation, and effector functions. These processes are energy-demanding, and recent studies have shown that miRNAs play a crucial role in regulating this dynamic metabolic reprogramming. By modulating the expression of metabolic genes, miRNAs influence both innate and adaptive immunity, shaping immune cell function in both health and disease [190].

When immune cells are activated, they undergo a metabolic shift from oxidative phosphorylation to glycolysis to ensure they have the necessary energy to execute their functions during immune activation. This process is enhanced by miRNAs like miR-155 and miR-21, as they promote pro-inflammatory responses in T cells and macrophages [191]. However, the role of miRNAs is broader, as they also regulate mitochondrial function, which is central to immune cell survival and differentiation. For instance, miR-181a and miR-23a impact mitochondrial oxidative metabolism, influencing T cell survival and differentiation, while miR-143 restricts glucose uptake in T cells by targeting glucose transporters, favoring memory cell formation [192].

Furthermore, immune cell activation is tightly controlled by the metabolism of amino acids such as glutamine and arginine. miRNAs such as miR-150 and miR-142 modulate the transport of these amino acids, thus affecting immune cell fate decisions [193]. Members of the let-7 family support oxidative phosphorylation (OXPHOS) in naïve T cells and shift macrophages toward a more oxidative phenotype via inhibition of AKT/mTOR [194].

In macrophages, miRNAs such as miR-21, miR-30c, and miR-125a-5p shape polarization and metabolic activity through controlling the PI3K/Akt, mTOR, and REDD1 pathways [195,196,197]. Furthermore, exosomal miRNAs, including miR-146a/b, miR-155, and let-7e, transferred from tumor cells to monocytes, drive differentiation into metabolically suppressed, immunosuppressive MDSCs [90].

**Figure 2:**
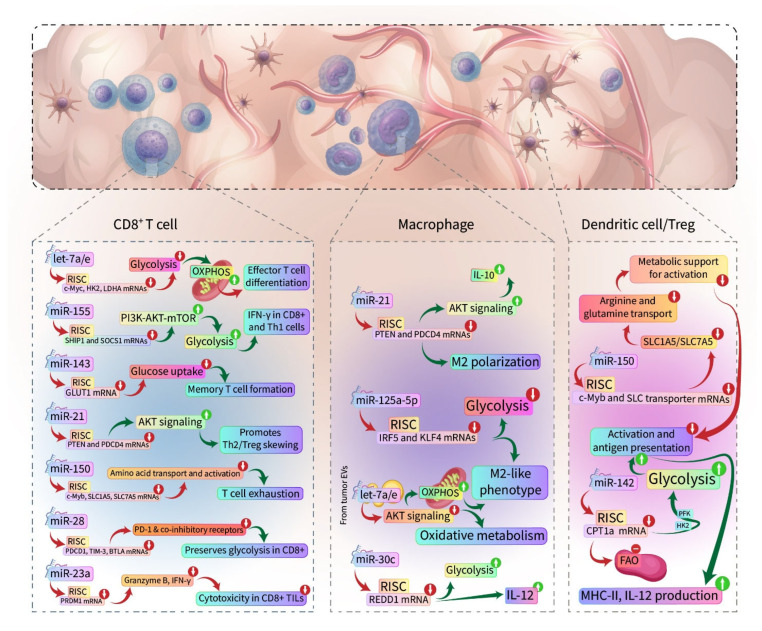
miRNA-mediated regulation of immunometabolism in the tumor microenvironment (TME). In CD8^+^ T cells, miRNAs such as let-7, miR-155, miR-143, and miR-21 modulate glycolysis, oxidative phosphorylation, glucose uptake, and amino acid transport, affecting effector differentiation, memory formation, and cytotoxicity. In macrophages, miR-21 and miR-125a-5p regulate AKT signaling, glycolysis, and polarization toward M2-like phenotypes, while let-7 and miR-30c influence oxidative metabolism and IL-12 production. In DCs and Tregs, miR-150 and miR-142 impact amino acid transport, glycolysis, FAO, and antigen presentation, collectively shaping immune activation and tolerance.

Together, these miRNAs integrate metabolic and immune signaling to shape the immune cell functions and promote a pro-tumorigenic TME (Table 2 and Figure 2).

## 6. Therapeutic Implications and Future Directions of miRNAs

miRNA-based therapies are a novel and rapidly advancing approach in cancer treatment, showing considerable promise by targeting gene expression pathways that drive tumor initiation, progression, and therapy resistance [201]. Several therapeutic strategies have been developed to exploit miRNA dysregulation in cancer, aiming either to restore tumor-suppressive miRNAs or inhibit oncogenic ones. Restoration of tumor-suppressive miRNAs is typically achieved using synthetic mimics. In contrast, overexpressed oncomiR can be silenced using chemically modified antisense oligonucleotides known as antagomiRs or anti-miRs, which have shown therapeutic potential across a range of cancer models. Additional approaches, such as miRNA sponges or decoys, can sequester multiple oncomiRs simultaneously, reducing their pathological activity. CRISPR/Cas-based genome editing offers another strategy by enabling permanent and precise modulation of miRNA genes. These interventions not only reprogram tumor behavior but also improve the efficacy of conventional therapies, such as chemotherapy, by overcoming resistance mechanisms [202,203].

**Figure 3:**
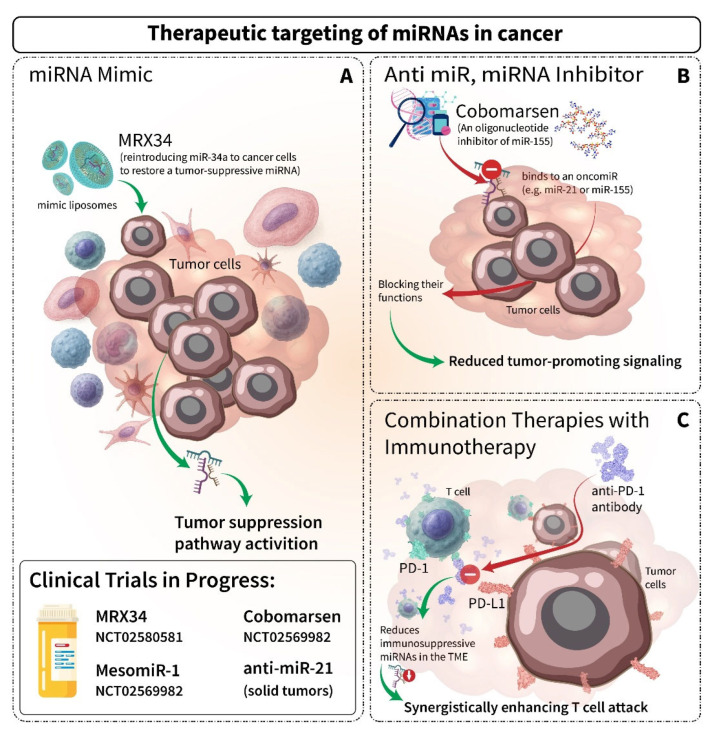
Therapeutic targeting of miRNAs in cancer and current strategies for miRNA-based cancer therapy. (**A**) miRNA mimics, such as MRX34 (a miR-34a mimic), are delivered to restore tumor-suppressive miRNA function and activate anti-tumor pathways. (**B**) Anti-miRs such as Cobomarsen (anti mir-21, miR-155) target oncomiRs to reduce tumor-promoting signaling. (**C**) Combination therapies such as anti-PD-1, to synergistically enhance T cell-mediated anti-tumor responses by reducing immunosuppressive miRNAs in the TME.

Clinical trials provide early validation of these strategies, while also highlighting key challenges in delivery and safety (Table 3 and Figure 3). Synthetic miRNA mimics have been developed to reintroduce tumor-suppressive miRNAs lost during disease progression. A notable example is miR-34a, which is delivered via liposomal nanoparticles (MRX34) in a Phase I trial for hepatocellular carcinoma, non-small cell lung cancer (NSCLC), and melanoma (NCT01829971) [204,205]. Although the trial demonstrated feasibility, it was halted due to immune-related toxicities, underscoring the need for improved delivery systems. Similarly, miR-16-based therapy (TargomiRs) was evaluated in patients with mesothelioma and NSCLC (NCT02369198), showing acceptable safety and early signs of clinical activity [206]. More recently, using lipid nanoparticle delivery, miR-193a-3p entered clinical evaluation for melanoma (NCT04675996).

Focusing on inhibition, anti-miRs aim to silence overactive oncomiR. Cobomarsen, a Locked Nucleic Acid (LNA)-modified anti-miR-155, showed disease-modifying activity in a Phase II trial for cutaneous T cell lymphoma (NCT02580552), validating miR-155 as a therapeutic target [207,208]. Additional candidates, such as anti-miR-221 in hepatocellular carcinoma (NCT02716012) and anti-miR-21 in solid tumors (NCT04556981), further support the therapeutic potential of miRNA inhibition, although LNA-based systems require careful monitoring for off-target effects [209,210].

Despite these advances, effective delivery remains one of the most significant challenges in translating miRNA therapies to the clinic. Current strategies rely heavily on lipid nanoparticles (as used in MRX34), EGFR-targeted bacterial minicells (e.g., MesomiR-1), and LNA-modified oligonucleotides. However, challenges related to immune activation, scalability, and tumor-specific targeting persist. For instance, while miR-29b delivered via bacterial minicells has shown preclinical efficacy in lung cancer, it has yet to progress to clinical trials. Looking ahead, next-generation strategies may focus on biomarker-guided patient selection and combination regimens. miRNAs such as miR-200c are under investigation as potential chemosensitizers in ovarian cancer [211], awaiting clinical validation. Although further advancements in delivery technologies, patient stratification, and combination strategies will be essential, the integration of miRNA therapeutics with immunotherapies and precision oncology frameworks holds substantial potential to enhance treatment response and specificity.

**Table 3:** Selected developments in clinical and preclinical miRNA-based cancer therapeutics.

miR (Drug Name)	RCT ID /Phase	Disease	Delivery Method	Key Findings/Status
Mimic				
**miR-34a** **(MRX34)**	NCT01829971 Phase I	Liver, lung, melanoma	Liposomal nanoparticles	The trial was halted due to immune toxicity, but validated miRNA mimic feasibility [204,205]
**miR-16** **(MesomiR-1)**	NCT02369198 Phase I	Mesothelioma, NSCLC	EGFR-targeted minicells	Safe, reduced tumor burden in some patients [206]
**miR-193a-3p**	NCT04675996 Phase I	Melanoma	Lipid nanoparticles	Ongoing trial testing safety and efficacy.
**miR-221**	NCT02716012 Phase I/II	HCC	LNA-modified antisense	Reduced miR-221 levels improved survival in HCC [209]
**miR-29b** **(TargomiRs)**	Preclinical	Lung cancer	Bacterial minicells	Promising preclinical results; no active trial yet [212]
**Anti-miR**				
**miR-155** **(Cobomarsen)**	NCT02580552 Phase II	CTCL	LNA-based antisense	Reduced disease activity in CTCL patients [207,208]
**miR-21**	NCT04556981 Phase I	Solid tumors	LNA antisense	Preclinical data show reduced metastasis; the trial is ongoing [210]

## 7. Conclusions

miRNA plays a pivotal role in shaping the cellular composition and functional dynamics of the TME by modulating immune cell behavior. They influence key immune subsets, including CTLs, NK cells, DCs, TAMs, and TANs. For example, miR-23a and miR-155 regulate CTL cytotoxicity and exhaustion, while miR-146a and miR-223 modulate macrophage and neutrophil polarization states. In the context of immune evasion, miRNAs such as miR-214, miR-21, and miR-125b promote the expansion and function of immunosuppressive populations, including Tregs and MDSCs, thereby attenuating anti-tumor immunity. In addition to their regulatory functions, several miRNAs serve as biomarkers of immune activity and tumor progression and represent promising therapeutic targets. They can enhance immunotherapy efficacy by boosting T or NK cell activation (e.g., miR-149-3p and miR-140-5p) or by directly targeting oncogenic signaling pathways and immune checkpoints (e.g., miR-34a and miR-326).

Furthermore, miRNAs hold potential for precision oncology as predictive biomarkers of response or resistance to immunotherapeutic agents. Therapeutic delivery of miRNA mimics or inhibitors, particularly when integrated with advanced delivery systems, offers a strategy to counteract immune suppression and reinvigorate immune surveillance within the TME. However, several barriers hinder clinical translation. A major challenge remains efficient, cell-specific delivery, while minimizing off-target effects and unintended immune activation. Most miRNA-based interventions remain in the preclinical stage, with limited clinical validation of targets and delivery platforms. Safety concerns, immunogenicity, and suboptimal pharmacokinetics further complicate development.

Future research should focus on elucidating the complex, cell-type-specific regulatory networks governed by miRNAs, including feedback loops and context-dependent effects. Integrative multi-omics approaches, combining miRNA, transcriptomic, proteomic, and metabolomic data, may identify actionable regulatory nodes. The development of miRNA-based combination therapies, such as co-administration with immune checkpoint inhibitors, metabolic modulators, or cancer vaccines, represents an emerging and promising frontier. Additionally, leveraging artificial intelligence and systems biology could accelerate the identification of miRNA targets and enhance predictive modeling for therapeutic responsiveness.

## Figures and Tables

**Table 1:** The role of miRNAs in the TME.

miRNA	Immune Cell/Target	Function/Effect	Context/Mechanism	Ref.
**miR-23a**	CD8^+^, NK	Suppresses effector functions, Impairs cytotoxicity, upregulated by TGF-β	Inhibits granzyme B, promotes immune evasion, transferred via exosomes under hypoxia	[11,12]
**miR-23b**	DC	Promotes tolerogenic DCs	Suppresses Notch1/NF-κB	[13,14]
**miR-139**	CD8^+^	Inhibits perforin expression, downregulation enhances cytotoxicity	Downregulated during inflammation	[15]
**miR-150**	CD8^+^, NK	Regulates differentiation into effector/memory subsets, maturation	Targets c-Myb, represses PI3K-AKT pathway	[15,16,17,18]
**miR-155**	CD8^+^, CD4^+^, DC, NK, TAM	Essential for CTL and Th1 responses, DC maturation, enhances NK cytotoxicity by upregulating NKG2D, IFN-γ, and granzyme B, drives M1 TAM, promotes NETosis by upregulating PAD4, enhances Treg function, modulate PD-L1, drives monocyte conversion to MDSC, and inhibits T cell exhaustion	Regulates interferon signaling, targets SOCS1, SHIP-1, C/EBPβ, c-Fos	[19,20,21,22,23,24,25,26,27,28]
**miR-194-5p**	Treg	Enhance function	Modulate PD-L1	[29]
**miR-135b**	CD8^+^, CD4^+^	Silencing reduces granzyme B and perforin, enhances IL-17 by repressing STAT6/GATA3	Silencing impairs cytotoxicity, promotes Th17	[30,31]
**miR-17-92 cluster**	CD8^+^, CD4^+^	CTL differentiation, supports Tfh function	Regulate CTL differentiation during antiviral and antitumor responses	[15,32,33]
**miR-326**	CD4^+^	Facilitates Th17 development	-	[32,33]
**miR-27a-5p**	CD4^+^, NK, DC	Inhibit Treg/Th17 and Th1 differentiation, Regulates NK chemotaxis	modulate CX3CR1 and CX3CL1, induced by TGF-β1, effects on chemotactic receptors and immunosuppressive ligands	[34]
**mir-27a/b**	TAM	Promote M1 polarization, pro-inflammatory	increased inflammatory cytokine production and tumoricidal activity	[23]
**miR-130a/b**	TAM, TAN, NK	Promote M1 polarization, pro-inflammatory, Controls neutrophil maturation, targets STAT3, enhance NK cytotoxicity	Regulates MPO, proteinase 3, TGF-β1, controlling cell cycle exit,	[23,35]
**miR-24**	CD4^+^, T cells	Promotes Th1/Th17, drives T cell exhaustion	immunosuppressive TME, Upregulates CD39, PD-1, TIM-3, dampens metabolism, targeting Myc and FGF11	[36]
**miR-107**	DC	Targets IL-23p19	alters T cell polarization	[37]
**let-7i**	DC	Suppresses immune response, suppresses SOCS1 and enhances DC activation	Alters cytokine expression, in response to LPS but not other TLR ligands	[38,39]
**miR-192**	Treg	Promotes Treg differentiation, enhances IL-10	Regulates via RB1/NF-κBp65	[40]
**miR-214**	Treg	Promotes Treg expansion, secretion of IL-10	Delivered from tumor cells via exosomes, by downregulating PTEN	[41]
**miR-34**	Treg, NK, Tumor	Suppresses CCL22, limits Treg recruitment, Upregulate ULBP2, enhance NK cytotoxicity	Downregulated by TGF-β, restoration reduces Treg infiltration, Tumor suppressors	[42,43,44]
**miR-223**	NK, TAN, TAM	Restrains NK cytotoxicity, regulates neutrophil maturation, NETosis, modulates cytokine secretion, promotes pro-metastatic behavior, and shapes cytokine milieu	Targets granzyme B, IGF-R, and influence TAM–tumor interactions	[25,26,27,45,46,47,48,49]
**miR-15/16**	NK, T cells	Targets IFN-γ, Bcl2, cyclin D1, restrains T cell function, restrains T cell function, and targets mTOR	Knockout enhances T cell responses, Knockout enhances proliferation, cytokine secretion, and T cell hyporesponsiveness	[50,51,52,53]
**miR-20a**	NK, CAF, TAM, TAN	Reduces MICA/B expression, suppresses PTEN/PI3K-AKT pathway, promotes angiogenesis, and neutrophil adhesion/migration	Upregulated in ovarian cancer, CAF-derived exosomes, target HIFs, and upregulate IL-8	[54,55]
**miR-29a**	NK, DC, TAM, MDSC	Inhibits melanoma, regulates B7-H3, Represses DNA methyltransferases, counters pro-inflammatory DC, enhances MDSC differentiation and function, in TAM creates a Treg/Th17 imbalance, and promotes immune suppression	Inhibits angiogenesis, modulates immune checkpoint, targets Hbp1, Prkar1a in MDSC, and STAT3 in CD4^+^	[56,57]
**miR-29b**	DC, TAM, Endothelial	Represses DNA methyltransferases, influencing epigenetic regulation and immune gene expression, regulates B7-H3, counters pro-inflammatory DC phenotype, inhibits angiogenesis, inhibits angiogenesis, and tumor growth	Inhibits melanoma, modulates immune checkpoint, targets Akt3, and inhibits VEGF/C-MYC	[58,59,60,61,62,63]
**miR-30e**	NK	Suppresses granzyme B and perforin in CD56dim NK cells, and is elevated under chronic IFN or IL-15 stimulation	Limits cytotoxicity, suppresses translation of PRF1	[64,65]
**miR-183**	NK	elevated in RCC, inhibits DAP12 (TYROBP), and impairs NK function	Upregulated by TGF-β	[66]
**miR-18a**	NK	Downregulates NKG2D and ligands, enhancing IFN-γ secretion and NK cell responses	Induced by IDO1, lncRNA-GAS5 counteracts	[67]
**miR-561-5p**	NK	Regulates NK cell recruitment	modulate CX3CR1 and CX3CL1, affects pulmonary metastasis	[34]
**miR-186**	NK	Inhibits neuroblastoma growth	Exosomal, suppresses immune escape	[68]
**miR-BART7**	NK	Downregulates MICA, reduces cytotoxicity	EBV-encoded	[69]
**miR-222,** **miR-339**	NK, T cells	Impairs cell interactions	Downregulate ICAM-1	[70]
**miR-148a**	DC	Inhibition enhances DC function	Targets DNMT1/SOCS1	[71]
**miR-148b-3p**	CAF	Reduced in CAF exosomes, enhances chemosensitivity	Suppresses Wnt/β-catenin, elevates PTEN	[72]
**miR-22**	DC, CAF, Treg	Impairs DC function, activates JAK/STAT3 in CAF and Tregs	Targets p38, promotes chemokine secretion (CCL17, CCL20, and CCL22), recruits Tregs via chemokines	[71,73]
**miR-212-3p**	DC	Inhibits MHC II expression and promotes tolerance	Taken up by DCs, inhibits RFXAP, and is delivered by pancreatic cancer exosomes	[74]
**miR-17-5p**	DC, Treg	Suppresses DC maturation and promotes Treg-mediated suppression	Upregulated in gastric cancer, and suppresses RUNX3	[75,76]
**miR-221**	DC	Regulates IL-12, apoptosis, and DC maturation	Targets p27kip1, KPC1, and SOCS1	[77]
**miR-5119**	DC	Enhances the immunogenicity of DCs by modulating checkpoint molecules	Improves responses in breast cancer	[78]
**miR-133a**	DC	As a tumor suppressor in osteosarcoma, and suppresses DC activation	Targets RBP-J	[79]
**miR-147**	TAM	Regulates TAM function, Attenuates inflammation	Target components of the TLR/NF-κB pathway	[71,80]
**miR-21**	TAN, TAM, CAF, MDSC	Modulate cytokine secretion, promote pro-metastatic behavior, shape cytokine milieu, promote expansion, activation, T cell inhibition, shape cytokine milieu, promote M2 TAM, and support the equilibrium, leading to immune evasion and increasing drug resistance	Induced by hypoxia, released by CAFs in exosomes, and stimulates MDSC generation via STAT3	[46,47,81]
**miR-21-5p**	TAM	Exosomes from TAMs create Treg/Th17 imbalance, and promote immune suppression and metastasis	Target STAT3 in CD4^+^	[57]
**miR-9**	TAM, MDSC, Endothelial	Overexpressed in tumors, skewed toward a tumor-promoting phenotype TAM, promote early development of MDSC via a pathway, and promote migration and angiogenesis	Target SOCS3, PIAS3 in Endothelial and JAK/STAT in MDSC, overexpressed in tumors, transferred from the tumor, and taken up by endothelial cells	[57,60,61,62,82]
**miR-1246**	TAM, MDSCs	Skewed toward tumor-promoting phenotype, transferred from the tumor, and promotes differentiation and activation	Shape TAM phenotypes via exosomal miRNAs, via DUSP3/ERK pathway	[60,61,62,83]
**miR-33**	TAM	Regulate the functional polarization of macrophages	Regulates lipid metabolism	[84]
**miR-17**	DC, Treg, TAM, TAN	Suppresses DC maturation, promotes Treg-mediated suppression, promotes angiogenesis, and neutrophil adhesion/migration	Upregulated in gastric cancer, suppresses RUNX3, targets HIFs, supports vascular remodeling in the TME, and upregulates IL-8	[17,55,76]
**miR-98,** **miR-720**	TAM	Regulate inflammatory cytokine balance and tumor cell migration	Modulate TAM-secreted	[85,86]
**miR-511-3p**	TAM	Limits pro-tumor function	Expressed in CD206^+^ TAMs	[87]
**miR-146a**	TAN, MDSC, NK, DC, TAN, Treg	Regulates NETosis, promotes immune suppression, suppresses NK proliferation/cytotoxicity, DC maturation, stabilizes Treg, promotes M2 TAM, and monocyte conversion to MDSC	Enhances IL-8, CCL5, induced by NF-κB, targets TRAF6/IRAK1,	[23,88,89,90]
**miR-146b**	NK, MDSC	Enhances NK activity against chemo-resistant cancer, and drives monocyte conversion to MDSC	Targets WBSCR22	[91,92]
**miR-1696**	TAN	Promotes NETosis, oxidative stress, and immune cell recruitment	Represses GPx3	[25,26,27,93]
**miR-16-5p**	TAN	NETosis-associated, regulates autophagy	Neutrophil survival and function in the tumor	[48,49]
**miR-4780**	TAN	Define N2 pro-tumorigenic phenotype	Upregulated in colon cancer, Target TUSC1	[94]
**miR-3938**	TAN	Define N2 pro-tumorigenic phenotype	Downregulated in colon cancer, targets ZNF197	[94]
**miR-138**	Tumor, NK, TAN	Modulates PD-L1/PD-1 signaling in tumor and immune cells, and suppresses neutrophil-derived NGAL	Reduces proliferation/metastasis in pancreatic cancer	[95,96]
**let-7b**	TAN, Tumor	Anti-inflammatory, suppresses TLR4, IL-6, IL-8, TNF-α, and suppresses tumor proliferation	Upregulates IL-10, can be delivered in exosomes, secreted via NK exosomes enriched	[97]
**miR-31**	TAN, CD8^+^	Promote neutrophil adhesion, promote CD8^+^ exhaustion, and upregulate PD-1, LAG3 and IL-10	Upregulating IL-8, induced by TCR activation, increases the transcription factor c-Maf and the PGE₂ receptor Ptger2	[17,36,98,99]
**miR-199, miR-722**	TAN	Limits immune surveillance	Inhibits neutrophil chemotaxis and TAN infiltration	[100,101]
**miR-142-3p**	TAN	Regulates neutrophil–macrophage interaction	Modulates TNF-α and inhibits PKC-α	[38,102]
**miR-125a**	TAM, MDSC	Contribute to PMN-MDSC development, reinforce macrophage-mediated suppression, and enrich in M2 TAMs	Drive monocyte conversion to MDSC. miR-125a redirects TAMs towards an M1	[23,88,89,90,103,104]
**miR-125b**	TAM, Treg, MDSC	Inhibit Treg proliferation, induce tumor-promoting TAM, increase inflammatory cytokines, promote M1 differentiation, and drive monocyte conversion to MDSC	Targets type I IFN pathway, downregulates TNFR2, FoxP3, targets LIPA, Tilt equilibrium by weakening Treg-mediated immunosuppression	[13,14,23,90,105]
**miR-28**	T cells	Suppresses checkpoint molecules, revives T cell activity	Downregulated in exhausted CD8^+^, and suppresses the checkpoint molecules PD-1, TIM-3, and BTLA	[106]
**miR-200c**	Tumor, T cells, Endothelial	Reduce PD-L1, reinvigorate T cells, inhibit migration, and angiogenesis	Suppressed in HBV-driven HCC via TGF-β/STAT3 signaling,	[82,107]
**miR-4772-3p**	Treg	Promotes Treg proliferation	Upregulates Helios (IKZF2)	[108]
**miR-128-3p**	Treg	Influences Treg enrichment in gastric cancer, promoting immune evasion	Interacting with IL16	[109]
**miR-182**	Treg	Promotes Treg polarization and formation of IL-17-producing Tregs, facilitating immune evasion	Targets FOXO1, NFATs, Increases FOXP3, TGF-β	[110]
**miR-325-3p**	Treg	Regulates Treg function in OSCC	Stabilizes Foxp3	[111]
**miR-208b**	Treg	Promotes expansion, chemoresistance	Targets PDCD4	[112]
**miR-423-5p**	Treg	Supports Treg-mediated immune evasion in HCC	Interacts with FENDRR	[113]
**miR-429**	Treg	Reduces Treg infiltration in HCC	Targets PD-L1	[114]
**miR-210**	MDSC	Promote expansion, MDSC activation, and T cell inhibition	Induced by hypoxia	[115,116]
**miR-92a**	MDSC	Enhance MDSC differentiation, function, and establishment of an immunosuppressive environment	Target Hbp1 and Prkar1a	[56]
**miR-1298-5p**	MDSC	Enhances immunosuppressive effects	Inhibits glioma progression	[117]
**miR-181a/b**	MDSC, NK, TAM	Promote early development via JAK/STAT pathway, enhance MDSC differentiation, immune suppression, tumor progression, promote IFN-γ in NK, and favor M2 polarization	Target SOCS3, PIAS3, NLK	[17,18,57]
**miR-181d-5p**	CAF	Inhibits CDX2, HOXA5, promotes EMT, and invasion in breast cancer	Taken up by cancer cells	[118]
**miR-494**	MDSC, Endothelial	Reduces immunosuppressive effects, promotes angiogenesis, and evasion	regulated by TGF-β and upregulated in gastric cancer, overexpressed in tumors	[119]
**miR-375**	TAM	Enhances migration and infiltration	Released from the apoptotic tumor, targets TNS3 and PXN in TAM and CCL2 in the tumor	[120]
**miR-101**	TAM	Facilitates M1→M2 transition, enhances proliferation/migration	Targets C/EBPα, KLF6, in breast and ovarian cancer	[121]
**miR-100**	TAM, MDSC	Sustains pro-tumor characteristics, influencing the mTOR pathway, driving monocyte conversion to MDSC	Linked to Stat5a/IL-1ra pathway, Links PGE2 and IL-6 signaling	[90,122]
**miR-149**	CAF, T cells	Immune evasion and tumor progression, reinvigorates T cells, and reverses exhaustion by targeting PD-1, TIM-3, BTLA, and Foxp1	Silenced to enhance IL-6 secretion in gastric cancer, links PGE2, and promotes EMT	[123,124,125]
**miR-500a-5p**	CAF	Targets USP28, facilitates proliferation, metastasis, immune evasion, and dissemination	Delivered via exosomes and taken up by cancer cells	[126]
**miR-369**	CAF	Enhances migration/invasion	Stimulates the MAPK pathway	[127]
**miR-196a**	CAF	Promotes migration/invasion, enhances CCL2 secretion	Targets ANXA1	[128]
**miR-196b**	Endothelial, NK	Upregulated by VEGF and promote angiogenesis	Targeted to reduce tumor growth	[129]
**miR-10b**	Endothelial	Upregulated by VEGF, promotes angiogenesis in breast tumors, downregulates MICB, a stress ligand for NKG2D	Targeted to reduce tumor growth and facilitate tumor escape	[129,130]
**miR-10a**	CD8^+^, DC, MDSC	T cell inhibition, influences cytotoxic molecule expression, targets IL-12/IL-23p40 in DC and RORA in glioblastoma, promotes expansion, activation, and alters the MDSCs via NF-κB	Downregulates antigen presentation induced by hypoxia	[115,116,131]
**miR-141**	Endothelial, Treg	Enhances angiogenesis and migration via JAK/STAT3, NF-Κb in ovarian cancer, recruits Tregs via CXCL1, and enhances angiogenesis	Released in extracellular vesicles, Activates JAK/STAT3, NF-κB	[109,132]
**miR-1468-5p**	Endothelial, T cell	Enhances PD-L1, promotes lymphangiogenesis, and causes immune escape	Activates JAK2/STAT3, secreted by cancer exosomes	[133]
**miR-526b, miR-655**	Endothelial	Promotes angiogenesis, lymphangiogenesis in breast cancer, upregulates VEGF, and downregulates PTEN	Induced by COX-2	[134]
**miR-200 and miR-128**	Endothelial	Inhibits migration and angiogenesis	Downregulated in tumors	[82]
**miR-103**	Endothelial	Enhances tumor cell death and inhibits angiogenesis	Exacerbates DNA damage	[135]

**Table 2:** miRNAs regulating immunometabolism in cancer.

miRNA	Target Immune Cell/Pathway	Mechanism of Action	Ref.
**let-7 family**	CD8^+^ (naïve → effector switch); TAMs via exosomes (AKT/mTOR)	In T cells, it suppresses c-Myc and glycolytic enzymes (e.g., HK2 and LDHA), maintaining OXPHOS in naïve cells. In TAMs, tumor exosomal let-7a inhibits AKT/mTOR, shifting macrophages to OXPHOS metabolism.	[194]
**miR-155**	CD8^+^ (PI3K/AKT/mTOR); Th1 cells; M1 Macrophages	Targets SHIP1, SOCS1 to unleash PI3K-AKT-mTOR signaling—increases glycolysis, proliferation, and IFN-γ in T cells. Inhibits IL-4/c-Maf to skew TH toward Th1 In macrophages, it promotes M1 polarization via JAK/STAT and NF-κB pathways.	[62,194,198]
**miR-143**	CD8^+^ (glucose metabolism via GLUT1)	Directly targets the GLUT1 transporter, reducing glucose uptake and glycolysis in T cells. Favors mitochondrial metabolism, supporting central memory T cell formation.	[194]
**miR-23a**	CD8^+^ (BLIMP-1, effector TFs)	TGF-β-induced in TILs; targets PRDM1 (BLIMP-1) and other effector genes. Reduces granzyme B, TNF, and IFN-γ in CD8^+^. Reduces cytotoxicity without affecting proliferation.	[11]
**miR-146a**	T cells (NF-κB/STAT1, checkpoints); Tregs; TAMs (TLR/NF-κB)	In T effector cells: targets IRAK1, TRAF6, STAT1, etc., dampening NF-κB and IFN pathways—leads to increased PD-1, CTLA-4, TIM-3, and LAG-3 on T cells (via indirect c-Fos effects). In Tregs, sustains suppressive function (prevents Th1 conversion). In macrophages, it reduces TLR signaling, curbing pro-IL-12/IL-1β and encouraging IL-10.	[199]
**miR-21**	Tumor-Associated Macrophages (PTEN/PI3K-Akt); CD4 T cells (indirect); MDSCs	Targets PTEN and PDCD4 in macrophages, activating PI3K/AKT and downregulating IL-12, promoting M2 polarization. TAM-derived exosomal miR-21 delivers these signals to neighboring cells, and also prevents apoptosis in tumor cells (via PTEN→AKT). In CD4^+^ T (induced by NF-κB), aids Th2 and Treg responses (resolves inflammation).	[195,196]
**miR-30c**	Macrophages (REDD1/mTOR axis in TAMs)	Targets REDD1, a negative regulator of mTOR. miR-30c keeps mTOR active, sustaining glycolysis and M1 phenotype. Hypoxia lowers miR-30c, resulting in REDD1 accumulation and mTOR inhibition (glycolysis ↓).	[194]
**miR-125a-5p**	Macrophages (IRF5, KLF4 pathways); TAMs in late-stage tumors	Likely targets transcripts (e.g., IRF5, a driver of M1; KLF4, influences M2), thereby shifting the balance to M2. (miR-125a also targets HK2 in tumor cells, but in TAMs it is linked to polarization). Induced by tumor cytokines.	[197]
**miR-142**	Dendritic Cells (CPT1a, metabolic switch); also Tregs (SOCS1)	In DCs, targets CPT1a to suppress FAO, enabling glycolysis and DC activation. Without miR-142, DCs remain oxidative (tolerogenic). In Tregs, targets SOCS1, promoting IL-2 signaling and Treg survival.	[198,200]
**miR-28**	CD8^+^ (PD-1 and co-inhibitory receptors)	Directly binds PDCD1 (PD-1) mRNA 3′UTR, preventing its translation. Also reported to downregulate other exhaustion markers (Tim-3 and BTLA). Preserves T cell glycolytic capacity and cytokine secretion by keeping the checkpoint low.	[106]
**miR-146a/b, miR-155, miR-125b, miR-100, let-7e (tumor EV cluster)**	Circulating Monocytes → MDSC differentiation (multiple targets)	These miRNAs are released in melanoma exosomes and taken up by CD14^+^ monocytes. Collectively, they reprogram transcription to induce an immunosuppressive, metabolically quiescent MDSC phenotype (increased Arg1, IDO, and nitric oxide). let-7e and miR-125b reduce pro-inflammatory mediators; miR-146a/b and miR-155 modulate NF-κB.	[90]

## Data Availability

Not applicable.

## List of Abbreviations

AGO, Argonaute; CAF, Cancer-Associated Fibroblast; DC, Dendritic Cells; DGCR8, DiGeorge syndrome critical region 8; EMT, epithelial–mesenchymal transition; H3K27me3, histone H3 lysine 27 trimethylation; IL, Interleukin; MiRISC, miRNA-induced silencing complex; MDSC, Myeloid-Derived Suppressor Cell; MiRNAs, MicroRNAs; NK, Natural Killer; NSCLC, non-small-cell lung cancer; OncomiR, Oncogenic miRNAs; RISC, RNA-induced silencing complex; Treg, Regulatory T Cell; TAM, Tumor-Associated Macrophage; TAN, Tumor-Associated Neutrophils; TME, Tumor Microenvironment; TGS, transcriptional gene silencing; UTR, untranslated region.
